# Supplementary material for: Well-Being, Mental Health, and Study Characteristics of Medical Students before and during the Pandemic
Source: Behav Sci (Basel). 2023 Dec 21;14(1):7. doi: 10.3390/bs14010007 (PMC10812729; doi:10.3390/bs14010007)
Supplement: Supplementary file 1 [file behavsci-14-00007-s001.zip › behavsci-2574899-supplementary.pdf]

Table S1. Differences in the cross sectional sample.

Variable	Sample size pre (*N*)	Sample size peri (*N*)	*T*-value	*p*-value	Mean pre	SD pre	Mean peri	SD peri	Scale range
PWB	373	376	1.74	.082	3.95	.42	3.90	.42	1-5
SWB	373	376	.65	.517	3.88	.71	3.85	.75	1-5
CIT	373	376	1.54	.125	3.94	.44	3.89	.45	1-5
EE	384	360	3.62	<.001	2.87	1.09	2.59	1.02	0-6
SA	400	369	-.25	.807	3.88	.64	3.89	.62	1-5
CD	400	369	3.15	.002	4.10	.74	3.93	.75	1-5
FBL	400	369	.71	.476	2.32	.93	2.27	.91	1-5
AUT	400	369	-1.25	.213	3.62	.94	3.69	.77	1-5
PT	400	369	-1.80	.072	1.74	.80	1.84	.75	1-5
OS	400	369	5.60	<.001	2.92	.82	2.60	.78	1-5
WO	400	369	7.17	<.001	3.37	.95	2.90	.87	1-5
IP	400	369	3.66	<.001	2.92	.99	2.66	.94	1-5
SSL	400	368	-3.97	<.001	2.54	.84	2.77	.80	1-5
SSC	400	368	.76	.449	4.17	.68	4.13	.72	1-5
SSP	400	368	.40	.689	4.35	.75	4.33	.72	1-5
SST	400	368	-2.31	.021	3.35	.57	3.45	.61	1-5
SS	492	360	-3.44	.001	3.66	.59	3.81	.61	1-5

PWB = psychological well-being, SWB = subjective well-being, CIT = thriving, EE = emotional exhaustion, SA = skill adequacy, CD = cognitive demands, FBL = feedback of lecturers, AUT = autonomy, PT = participation, OS = organizational stressors, WO = work overload, IP = information problems, SSL = social support of lecturers, SSC = social support of colleagues, SSP = social support private, SST = social support total, SS = study satisfaction

Table S2. Differences in the cross sectional sample, divided by sex.

Men										Women									
Variable	Sample size pre (*N*)	Sample size peri (*N*)	*T*-value	*p*-value	Mean pre	SD pre	Mean peri	SD peri	Scale range	Variable	Sample size pre (*N*)	Sample size peri (*N*)	*T*-value	*p*-value	Mean pre	SD pre	Mean peri	SD peri	Scale range
PWB	156	149	-.01	.994	3.94	.42	3.94	.40	1-5	PWB	217	227	2.21	.028	3.95	.42	3.86	.42	1-5
SWB	156	149	-.32	.749	3.90	.70	3.92	.74	1-5	SWB	217	227	1.06	.291	3.87	.72	3.80	.75	1-5
CIT	156	149	-.09	.925	3.94	.44	3.94	.44	1-5	CIT	217	227	2.01	.045	3.94	.45	3.85	.45	1-5
EE	157	144	3.24	.001	2.92	1.13	2.51	1.07	0-6	EE	227	216	1.96	.050	2.84	1.06	2.64	.98	0-6
SA	162	148	-1.18	.238	3.91	.65	3.99	.62	1-5	SA	238	221	.66	.508	3.87	.64	3.83	.61	1-5
CD	162	148	2.51	.013	4.05	.73	3.83	.79	1-5	CD	238	221	2.03	.043	4.14	.74	4.00	.71	1-5
FBL	162	148	-.13	.897	2.37	1.00	2.38	.95	1-5	FBL	238	221	1.07	.284	2.28	.89	2.20	.87	1-5
AUT	162	148	-1.70	.090	3.59	.92	3.76	.82	1-5	AUT	238	221	-.20	.841	3.63	.95	3.65	.74	1-5
PT	162	148	-2.41	.016	1.73	.81	1.96	.85	1-5	PT	238	221	-.24	.809	1.75	.80	1.76	.67	1-5
OS	162	148	4.61	< .001	3.00	.79	2.59	.78	1-5	OS	238	221	3.48	.001	2.87	.84	2.61	.77	1-5
WO	162	148	4.48	< .001	3.31	.98	2.83	.89	1-5	WO	238	221	5.60	<.001	3.41	.93	2.95	.85	1-5
IP	162	148	1.63	.105	2.90	1.01	2.72	.97	1-5	IP	238	221	3.42	.001	2.93	.98	2.63	.93	1-5
SSL	162	148	-2.94	.004	2.56	.85	2.85	.84	1-5	SSL	238	220	-2.71	.007	2.52	.83	2.72	.77	1-5
SSC	162	148	.35	.728	4.14	.65	4.11	.74	1-5	SSC	238	220	.70	.487	4.18	.70	4.14	.71	1-5
SSP	162	148	-.01	.991	4.25	.79	4.25	.73	1-5	SSP	238	220	.56	.577	4.42	.71	4.38	.70	1-5
SST	162	148	-1.83	.068	3.35	.58	3.48	.65	1-5	SST	238	220	-1.47	.143	3.35	.57	3.43	.58	1-5
SS	187	144	-3.03	.003	3.65	.64	3.86	.63	1-5	SS	305	216	-1.89	.060	3.67	.56	3.77	.59	1-5

PWB = psychological well-being, SWB = subjective well-being, CIT = thriving, EE = emotional exhaustion, SA = skill adequacy, CD = cognitive demands, FBL = feedback of lecturers, AUT = autonomy, PT = participation, OS = organizational stressors, WO = work overload, IP = information problems, SSL = social support of lecturers, SSC = social support of colleagues, SSP = social support private, SST = social support total, SS = study satisfaction

Table S3. Differences in the cross sectional sample, divided by study years.

	Variable	Sample size pre (*N*)	Sample size peri (*N*)	*T*-value	*p*-value	Mean pre	SD pre	Mean peri	SD peri	Scale range
**Year 1**	PWB	216	130	1.41	.159	3.99	.41	3.92	.41	1-5
	SWB	216	130	1.70	.090	3.92	.67	3.79	.73	1-5
	CIT	216	130	1.56	.120	3.98	.43	3.90	.44	1-5
	EE	220	124	2.19	.029	2.75	1.04	2.50	1.01	0-6
	SA	231	127	1.39	.164	3.93	.62	3.83	.61	1-5
	CD	231	127	1.13	.260	4.19	.69	4.10	.64	1-5
	FBL	231	127	.53	.594	2.42	.95	2.36	.96	1-5
	AUT	231	127	-.18	.858	3.74	.85	3.76	.77	1-5
	PT	231	127	-.34	.737	1.77	.76	1.80	.77	1-5
	OS	231	127	3.47	.001	2.76	.80	2.46	.74	1-5
	WO	231	127	3.22	.001	3.29	.93	2.99	.78	1-5
	IP	231	127	1.75	.081	2.77	1.00	2.58	.94	1-5
	SSL	231	127	-2.83	.005	2.66	.79	2.91	.84	1-5
	SSC	231	127	.31	.754	4.20	.63	4.18	.67	1-5
	SSP	231	127	-.18	.854	4.34	.74	4.36	.68	1-5
	SST	231	127	-1.74	.083	3.43	.54	3.54	.63	1-5
	SS	281	124	-1.44	.151	3.83	.56	3.92	.63	1-5
	**Variable**	**Sample size pre (*N*)**	**Sample size peri (*N*)**	***T*-value**	***p*-value**	**Mean pre**	**SD pre**	**Mean peri**	**SD peri**	**Scale range**
**Year 2**	PWB	73	121	-.49	.627	3.89	.42	3.92	.44	1-5
	SWB	73	121	-1.20	.232	3.82	.79	3.95	.74	1-5
	CIT	73	121	-.71	.478	3.88	.45	3.93	.47	1-5
	EE	76	121	2.11	.036	3.02	1.15	2.71	.89	0-6
	SA	78	121	-.90	.367	3.87	.60	3.95	.54	1-5
	CD	78	121	3.04	.003	4.22	.66	3.90	.77	1-5
	FBL	78	121	-.97	.334	2.17	.87	2.29	.91	1-5
	AUT	78	121	-2.21	.029	3.49	.94	3.77	.78	1-5
	PT	78	121	-2.76	.006	1.54	.78	1.85	.75	1-5
	OS	78	121	4.82	< .001	3.20	.74	2.66	.79	1-5
	WO	78	121	5.70	< .001	3.62	.91	2.88	.88	1-5
	IP	78	121	3.46	.001	3.15	.85	2.69	.95	1-5
	SSL	78	121	-3.78	< .001	2.32	.88	2.77	.8	1-5
	SSC	78	121	1.39	.167	4.16	.77	4.00	.83	1-5
	SSP	78	121	.14	.886	4.28	.82	4.26	.73	1-5
	SST	78	121	-1.56	.121	3.24	.64	3.39	.66	1-5
	SS	98	121	-3.99	< .001	3.53	.54	3.82	.55	1-5
		**Sample size pre (*N*)**	**Sample size peri (*N*)**	***T*-value**	***p*-value**	**Mean pre**	**SD pre**	**Mean peri**	**SD peri**	**Scale range**
**Year 3**	PWB	40	68	-.20	.843	3.86	.47	3.88	.38	1-5
	SWB	40	68	.10	.921	3.79	.76	3.78	.76	1-5
	CIT	40	68	-.14	.893	3.85	.48	3.86	.41	1-5
	EE	41	62	1.93	.056	2.98	1.06	2.52	1.23	0-6
	SA	41	66	-1.06	.293	3.82	.64	3.96	.68	1-5
	CD	41	66	-.12	.904	3.87	.82	3.89	.77	1-5
	FBL	41	66	1.09	.276	2.33	.89	2.14	.91	1-5
	AUT	41	66	-.38	.707	3.65	.90	3.71	.72	1-5
	PT	41	66	.45	.658	1.85	1.01	1.77	.74	1-5
	OS	41	66	1.49	.139	2.95	.80	2.72	.79	1-5
	WO	41	66	3.35	.001	3.48	.84	2.86	.97	1-5
	IP	41	66	.30	.763	2.88	.98	2.82	1.01	1-5

	Variable	Sample size pre (*N*)	Sample size peri (*N*)	*T*-value	*p*-value	Mean pre	SD pre	Mean peri	SD peri	Scale range
	SSL	41	65	-.40	.691	2.53	.91	2.59	.79	1-5
	SSC	41	65	-2.20	.030	3.94	.74	4.24	.62	1-5
	SSP	41	65	-.36	.717	4.39	.67	4.44	.72	1-5
	SST	41	65	-1.57	.119	3.24	.60	3.42	.56	1-5
	SS	57	62	-2.45	.016	3.49	.55	3.75	.62	1-5
	**Variable**	**Sample size pre (*N*)**	**Sample size peri (*N*)**	***T*-value**	***p*-value**	**Mean pre**	**SD pre**	**Mean peri**	**SD peri**	**Scale range**
**Year 4**	PWB	32	37	.97	.335	3.92	.47	3.82	.42	1-5
	SWB	32	37	-.27	.786	3.83	.76	3.88	.76	1-5
	CIT	32	37	.68	.497	3.91	.50	3.83	.45	1-5
	EE	33	35	3.07	.003	3.22	1.17	2.38	1.08	0-6
	SA	35	36	-.74	.460	3.79	.81	3.92	.58	1-5
	CD	35	36	.87	.385	3.75	.76	3.58	.86	1-5
	FBL	35	36	-.59	.559	1.98	.92	2.10	.81	1-5
	AUT	35	36	.09	.927	3.27	1.16	3.25	.78	1-5
	PT	35	36	-.55	.581	1.80	.83	1.90	.73	1-5
	OS	35	36	3.62	.001	3.29	.85	2.56	.86	1-5
	WO	35	36	2.43	.017	3.32	1.07	2.75	.9	1-5
	IP	35	36	2.35	.021	3.19	1.02	2.64	.96	1-5
	SSL	35	36	-1.77	.081	2.49	.84	2.81	.67	1-5
	SSC	35	36	-.52	.601	4.15	.62	4.23	.65	1-5
	SSP	35	36	1.19	.239	4.46	.69	4.24	.84	1-5
	SST	35	36	-1.67	.099	3.32	.54	3.52	.47	1-5
	SS	37	35	-1.60	.114	3.32	.61	3.55	.63	1-5
	**Variable**	**Sample size pre (*N*)**	**Sample size peri (*N*)**	***T*-value**	***p*-value**	**Mean pre**	**SD pre**	**Mean peri**	**SD peri**	**Scale range**
**Year 5**	PWB	11	13	1.32	.202	3.97	.23	3.79	.39	1-5
	SWB	11	13	.63	.536	3.92	.61	3.74	.79	1-5
	CIT	11	13	1.16	.259	3.96	.27	3.78	.45	1-5
	EE	13	11	-.53	.599	2.72	1.34	2.98	.97	0-6
	SA	14	12	-.43	.668	3.59	.84	3.73	.79	1-5
	CD	14	12	-1.31	.202	3.48	.93	3.92	.75	1-5
	FBL	14	12	-.01	.991	2.36	1.07	2.36	.59	1-5
	AUT	14	12	-1.56	.134	3.05	1.28	3.67	.66	1-5
	PT	14	12	-1.28	.214	1.75	.94	2.17	.69	1-5
	OS	14	12	.50	.621	3.01	.81	2.87	.68	1-5
	WO	14	12	.80	.434	3.04	1.13	2.71	.93	1-5
	IP	14	12	2.75	.012	3.43	1.02	2.56	.56	1-5
	SSL	14	12	-.80	.433	2.05	.75	2.25	.49	1-5
	SSC	14	12	.76	.457	4.19	.86	3.97	.54	1-5
	SSP	14	12	.43	.668	4.45	.79	4.33	.57	1-5
	SST	14	12	.04	.970	3.12	.62	3.11	.42	1-5
	SS	17	11	-1.26	.217	3.11	.54	3.35	.4	1-5

PWB = psychological well-being, SWB = subjective well-being, CIT = thriving, EE = emotional exhaustion, SA = skill adequacy, CD = cognitive demands, FBL = feedback of lecturers, AUT = autonomy, PT = participation, OS = organizational stressors, WO = work overload, IP = information problems, SSL = social support of lecturers, SSC = social support of colleagues, SSP = social support private, SST = social support total, SS = study satisfaction

Table S4. Differences between study years in the COVID-specific questions.

Variable	(IV) How much impact do the pandemic containment measures have on your student life?					(V) I feel sufficiently informed about the Corona prevention measures at my university.					(X) Due to the lack of or limited attendance during my studies, I find it difficult to make new friends.				
	Compared study years	Sample Size (*N*)	Middle ranks	*U*-value	*p*-value	Compared study years	Sample Size (*N*)	Middle ranks	*U*-value	*p*-value	Compared study years	Sample Size (*N*)	Middle ranks	*U*-value	*p*-value
	1 vs. 2	1: 147<br>2: 130	12.46<br>159.97	6829.500	<.001	1 vs. 2	1: 146<br>2: 130	145.59<br>13.54	8455.500	.083	1 vs. 2	1: 142<br>2: 130	125.54<br>148.48	7673.000	.012
	1 vs. 3	1: 147<br>3: 91	107.46<br>138.96	4918.000	<.001	1 vs. 3	1: 146<br>3: 91	129.36<br>102.38	513.500	.001	1 vs. 3	1: 142<br>3: 90	112.18<br>123.31	5777.000	.199
	1 vs. 4	1: 147<br>4: 53	91.88<br>124.42	2628.000	<.001	1 vs. 4	1: 146<br>4: 53	101.90<br>94.77	3592.000	.387	1 vs. 4	1: 142<br>4: 52	91.04<br>115.15	2774.000	.006
	1 vs. 5	1: 147<br>5: 34	88.12<br>103.47	2075.000	.113	1 vs. 5	1: 146<br>5: 34	94.07<br>75.19	1961.500	.034	1 vs. 5	1: 142<br>5: 34	82.60<br>113.16	1575.5	.001
	1 vs. 6	1: 147<br>6: 23	83.37<br>99.09	1378.000	.144	1 vs. 6	1: 146<br>6: 23	87.23<br>7.83	1353.000	.094	1 vs. 6	1: 142<br>6: 22	82.21<br>84.34	1521.500	.838
	2 vs. 3	2: 130<br>3: 91	112.15<br>109.36	5765.500	.743	2 vs. 3	2: 130<br>3: 91	116.68<br>102.88	5176.000	.086	2 vs. 3	2: 130<br>3: 90	113.25<br>106.53	5493.000	.422
	2 vs. 4	2: 130<br>4: 53	9.35<br>96.05	323.500	.497	2 vs. 4	2: 130<br>4: 53	9.99<br>94.48	3313.500	.657	2 vs. 4	2: 130<br>4: 52	89.67<br>96.07	3142.5	.438
	2 vs. 5	2: 130<br>5: 34	84.95<br>73.12	1891.000	.183	2 vs. 5	2: 130<br>5: 34	84.38<br>75.31	1965.500	.279	2 vs. 5	2: 130<br>5: 34	79.91<br>92.41	1873.000	.152
	2 vs. 6	2: 130<br>6: 23	77.27<br>75.50	146.500	.857	2 vs. 6	2: 130<br>6: 23	78.36<br>69.33	1318.500	.327	2 vs. 6	2: 130<br>6: 22	78.01<br>67.57	1233.5	.283
	3 vs. 4	3: 91<br>4: 53	7.25<br>76.37	2206.500	.382	3 vs. 4	3: 91<br>4: 53	68.21<br>79.87	2021.000	.079	3 vs. 4	3: 90<br>4: 52	68.16<br>77.29	2039.000	.183
	3 vs. 5	3: 91<br>5: 34	64.99<br>57.68	1366.000	.302	3 vs. 5	3: 91<br>5: 34	62.84<br>63.44	1532.000	.929	3 vs. 5	3: 90<br>5: 34	58.91<br>72.00	1207.000	.059
	3 vs. 6	3: 91<br>6: 23	57.64<br>56.93	1033.5	.925	3 vs. 6	3: 91<br>6: 23	57.76<br>56.46	1022.5	.857	3 vs. 6	3: 90<br>6: 22	57.31<br>53.18	917.000	.579
	4 vs. 5	4: 53<br>5: 34	47.42<br>38.68	72.000	.105	4 vs. 5	4: 53<br>5: 34	46.49<br>4.12	769.000	.211	4 vs. 5	4: 52<br>5: 34	42.18<br>45.51	815.5	.522
	4 vs. 6	4: 53<br>6: 23	39.41<br>36.41	561.500	.577	4 vs. 6	4: 53<br>6: 23	4.19<br>34.61	52.000	.272	4 vs. 6	4: 52<br>6: 22	39.81<br>32.05	452.000	.137
	5 vs. 6	5: 34<br>6: 23	28.03<br>3.43	358.000	.583	5 vs. 6	5: 34<br>6: 23	29.35<br>28.48	379.000	.836	5 vs. 6	5: 34<br>6: 22	31.72<br>23.52	264.500	.053

Table S5. Differences in the longitudinal sample.

Variable Name	Sample size (*N*)	T - value	p - value
CIT _pre vs. CIT_peri	28	1.60	.121
PWB_pre vs. PWB_peri	28	2.02	.054
SWB_pre vs. SWB_pre	28	-.26	.795
EE_pre vs. EE_peri	30	.47	.643
SA_pre vs. SA_peri	32	-.66	.511
CD_pre vs. CD_peri	32	1.50	.143
FBL_pre vs. FBL_peri	32	1.20	.238
AUT_pre vs. AUT_peri	32	1.39	.175
PT_pre vs. PT_peri	32	1.16	.255
OS_pre vs. OS_peri	32	1.52	.139
WO_pre vs. WO_peri	32	3.15	.004
IP_pre vs. IP_peri	32	1.63	.112
SSL_pre vs. SSL_peri	32	.78	.440
SSC_pre vs. SSC_peri	32	.88	.384
SSP_pre vs. SSP_peri	32	.44	.665
SST_pre vs. SST_peri	32	1.03	.313
SS_pre vs. SS_peri	50	2.65	.011

Pre= before pandemic (2017-2020), peri = during pandemic (2022); CIT = thriving, PWB = psychological well-being, SWB = subjective well-being, EE = emotional exhaustion, SA = skill adequacy, CD = cognitive demands, FBL = feedback of lecturers, AUT = autonomy, PT = participation, OS = organizational stressors, WO = work overload, IP = information problems, SSL = social support of lecturers, SSC = social support of colleagues, SSP = social support private, SST = social support total, SS = study satisfaction
